# Repeated restraint stress induced neurobehavioral and sexual hormone disorders in male rats

**DOI:** 10.3934/Neuroscience.2022014

**Published:** 2022-05-25

**Authors:** Ahlem Matallah, Rabie Guezi, Abdelmadjid Bairi

**Affiliations:** 1 Department of Biology, Faculty of natural and life sciences, Kasdi Merbah Ouargla university, Algeria; 2 Laboratory Saharan Bio-Resources, Department of Biology, Faculty of natural and life sciences. Kasdi Merbah Ouargla university, Algeria; 3 Applied Neuroendocrinology Laboratory, Department of Biology, Faculty of Science, Badji Mokhtar Annaba University, Algeria

**Keywords:** CRS, depression, behavior, open field, ACTH, testosterone

## Abstract

Several studies have demonstrated that depression include disruptions not only for mental human disorders but also their healthy living. Rodent-based behavioral tests and models are widely used to understand the mechanisms by which stress triggers anxiety-related behaviors.

This present study examined the evidence of a chronic restraint stress (CRS) paradigm in male Wistar rats for the progressive nature of depression alongside with related changes in behavior and functions. The body weight was determined, and the behavior tests, including sucrose preference and the open field test were performed. Theses parameters confirme the presence of anxiety-like and depression-like behaviors beside that we will focus on the response of ACTH and testosterone concentrations in rats.

The results obtained during the experiment show that CRS led to decrease the time spent in the field center, a decrease of total distance travelled, in the stressed group compared with the control group. A significant increased of ACTH levels and decreased in testosterone hormone levels in the CRS. According to these results the CRS rodent model has value to validating the development for depression.

## 1. Introduction

Exposure to stress, especially at early ages, has been shown to be a determining factor in the appearance of depression and anxiety in humans [1]. Stress initiates a variety of physiological processes of the central and peripheral systems, typically leading to neuroendocrine responses, such as glucocorticoid (cortisol in humans and corticosterone in rodents) secretion, through the activation of the hypothalamic-pituitary-adrenal (HPA) axis [2]. As well, the release of corticosteroid and noradrenaline, which are caused by the activity of (HPA) axis and sympathetic nervous system (SNS), respectively, initiates a number of behavioral and physiological changes: irritable mood, loss of interest or pleasure, decreased energy and fatigue, low appetite or overeating, learning and memory [3],[4].

Moreover, glucocorticoids act on virtually all tissues to mobilize energy stores, enhance cognition, and increase cardiovascular activity, while suppressing immune, reproductive, and digestive functions [5],[6]. Unfortunately, sustained glucocorticoid increases caused by chronic stress might have negative health repercussions. Although, for study the etiology of human reproductive system disease, the researcher will be able to identify the biological mechanism of depression on the reproductive responses detected in animals, models of depression. This is performed through exposing them to stressful situations. In rodent models of chronic stress, physical restraint, which is one of the most widely used methods of animal model stress exposure, performed by placing mice into restraint apparatuses has been for several minutes to several hours (in most cases, for 2–6 hours) each day for a number of weeks (typically 1–3 weeks) [7],[8].

Several studies have been reported that CRS induced disturbance in reproductive functions, as well as induces suppression of testosterone secretion, libido and spermatogenesis in males [9].

Spermatogenesis is the key of sexual reproduction. It is straight forward to testicular physiology. This process depends on pituitary gonadotropins, follicle stimulant hormone (FSH) and luteinizing hormone (LH) as well as adequate testosterone hormone concentrations [10]. Evenly acute and chronic stress are effective to cause disturbance of male reproduction [11]. The hypothalamic-pituitary-gonadal (HPG) axis regulates reproduction. The two pituitary gonadotropins, luteinizing hormone (LH) and folliclestimulating hormone (FSH), are responsible for maintaining testicular testosterone (T) synthesis and spermatogenesis in males (FSH). The Leydig cells in the interstitial space are the LH target cells, while the Sertoli cells in the seminiferous tubules are the FSH target cells. LH stimulates Leydig cell T production, and FSH stimulates in Sertoli cells, in synergy with T, the production of regulatory molecules and nutrients needed for the maintenance of spermatogenesis [12]. In turn, the hypothalamus and pituitary gland could recognized the production of these hormones .Obviously, the principal regulator of the HPA axis, corticotrophin-releasing hormone (CRH) and its receptors are located in the ovaries, decidual endometrial stroma, placental trophoblast and even in the Leydig cells of the testis [13].

Hence, this current study was designed to examine the potential effects of chronic restraint stress on rat neurobehavior, besides the activity of the hypophysis-adrenal axis by determining the ACTH circulating levels and also the testosterone hormone levels to understand the fate of reproduction disability following HPA axis reactivity.

## 2. Materials and methods

### 2.1. Animals

20 male Wistar rats (250–300 g body weight; 10–11week-old), were purchased from the Pasteur institute of Algiers. They were housed ten per cage, all animals had free access to food and water, except for the chronic restraint stress (CRS) technique and fasting before the sucrose preference test, they were maintained in under standard conditions in a temperature-controlled room (23 ± 2 °C) and at 12 h/12 h light/dark cycle. Prior to the experiment, animals were weighted and divided into two groups, as follows: (1) control, (2) CRS. Animals in the control group were reared in single cages without any environmental stresses unlike animals in CRS were restrained in well ventilated tubes. Behavioral tests were carried out at baseline and after the last restraint procedure. Obviously, we are using the sucrose preference and open field tests as behaviors related to depression or anxiety in rats.

### 2.2. Chronic restraint stress procedure

**Figure 1.**
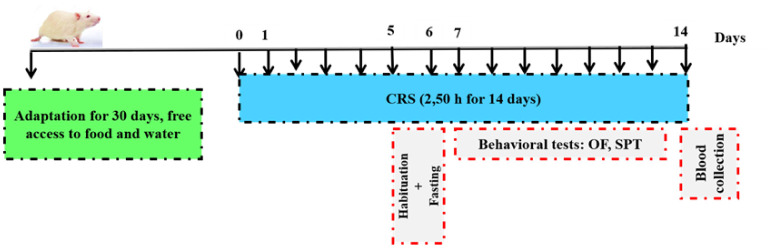
Schematic representation of the experimental design; the CRS stress protocol will be performed for 14 days.

The CRS was carried out as previously described [14] with minor modifications. Briefly, during the CRS paradigm, stressed rats were transported from the colony room to a quiet room and restrained. They were exposed for one time/day for 14 days at random hours, to avoid habituation. In brief, animals were placed in a transparent cylindrical restrainer (5 × 19 cm) with a nose-hole for ventilation, in which it is unable to move, for 2.5 h a day for 14 consecutive days [15]. Rat in the control group were left undisturbed in their home cages except when measuring body weight and SPT. Only in the last week, after each session of restraint stress, rats were subjected to OFT for 5 min. The body weight was determined throughout the experiment. The experimental design is depicted in (Figure 1).

This current study followed the guidelines of ethics on the animals used in experiments.

### 2.3. Body weight measurement

Rats' body weight was measured at the start of the experiment, and again after the last session of stress exposure.

### 2.4. Behavioral testing

#### 2.4.1. Anhedonia (sucrose preference test)

Anhedonia, is losing of interest in things were once pleasurable, it can be assessed by means of the sucrose preference test. Rats' animal experimentation model is based on the preference nature of water with sucrose dissolved in it over regular water [16],[17]. Wherein the preference pattern of sucrose solutions concentrations at 1% [18]. When a rodent shows a lack of interest in the sucrose solution, it is said to be exhibiting anhedonia which is a classic component of depressive disorders [19]. Sucrose preference was calculated as follows: succrose preference percentage (sucrose consumed/sucrose+ water consumed, %), to control for potential bias due to fluid intake.

#### 2.4.2. Open field test (OFT)

It's often used to assay general locomotor activity levels, anxiety and exploration. The open field apparatus (70 × 70 × 40 cm) was fabricated from white acrylic with a floor divided into 9 squares (10 × 10 cm each) by black lines. In OFT, the rodent is placed in a large circular or square enclosure and allowed to move ad-libitum [20].

The total distance traveled (cm), as well as frequency of entering the center were recorded for 5 min using video camera [21]. A non-anxious rodent will explore the center of the arena more than an anxious one, which will stick to the walls or only stay in the corner [22].

### 2.5. Biochemical assays

At the end of the experimental procedure (14 days), after the last restraint stress session was completed, blood samples of all animals were taken from jugular vein, and were collected in heparinized tube and in tube containing EDTA, then centrifuged with 5000 tr/min during 15 mn, the plasma separated kept at –20 °C till needed for analysis. The testosterone concentration was measured as previously described by the conventional ELISA method [23]. ACTH concentration calculated following the protocol provided by the manufacturer of the kit.

### 2.6. Statistical analysis

All results are expressed as mean ± standard error of the mean (SEM). Data and interactions were evaluated by one-way analysis of variance ANOVA was performed for multiple comparisons followed by student T for paired data. Analysis test was used for analysis of changes in body weight, on the number of behavioral measures of the test battery, ACTH and testosterone hormone level. *p* < 0.05 was required for results to be considered statistically significant. All analysis was performed using statistic Minitab version 16.0. The level of probability was set at (*p* < 0.05) as statistically significant.

## 3. Results

### 3.1. Body weight

**Figure 2.**
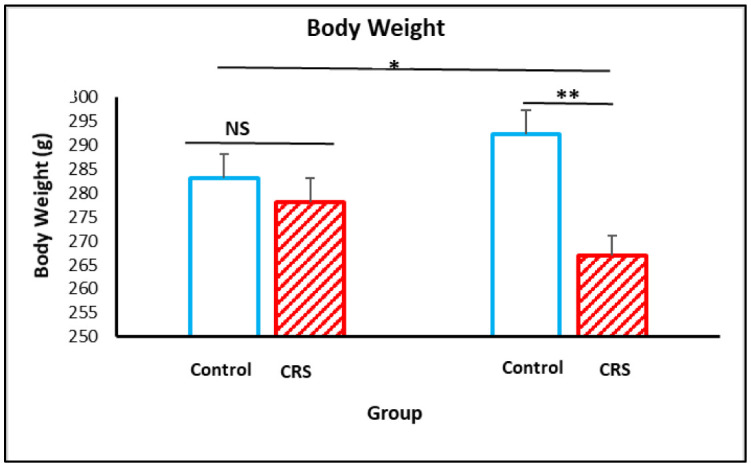
The effect of CRS on body weight. Body weight measured during CRS exposure. The results are expressed as the mean ± SEM (n = 10 per group). *p < 0.05; **p < 0.01, *** *p* < 0.001.

As shown in Figure 2, one way analysis of variance (ANOVA) of body weight revealed significant differences between the stress group and the control one. After 14 days of CRS procedure (Figure 2), rats in the CRS group had significant decreased body weight change after exposure (292.5 ± 5.2 versus 267.0 ± 4.7 g), (F = 4.37, *p* = 0.010) as compared to control group rats.

### 3.2. Anhedonia (sucrose preference test)

**Figure 3.**
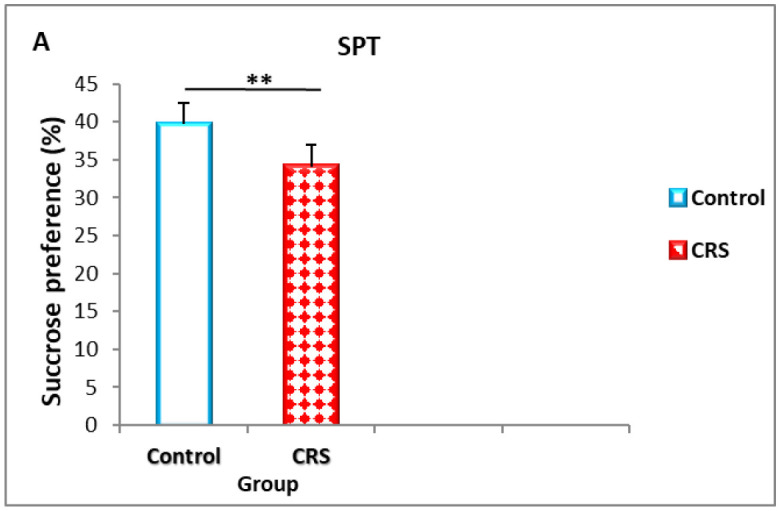
The effect of CRS on rat in the SPT. The results are expressed as the mean ± SEM (n = 10 per group). *p < 0.05; **p < 0.01, *** *p* < 0.001.

The SPT were performed to assess depressive-like behavioral changes. Following 14 days of CRS exposure (Figure 3), rats in the CRS group showed decreased sucrose consumption (%) as compared to the control group (39.7 ± 0.94 versus 34.10 ± 1.2), (*p* = 0.002).

### 3.3. Open field test

**Figure 4.**
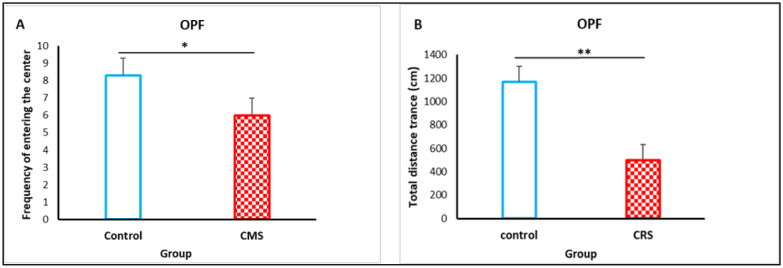
Effect of CRS on rat in the OFT. A, Frequency of entering the center. B, The total distance. The results are expressed as the mean ± SEM (n = 10 per group). *p < 0.05; **p < 0.01, *** *p* < 0.001.

The OFT test, were performed to assess anxiety-like behavioral changes in rat after CRS. In this test, we assessed the effect of chronic restraint stress on the frequency of entering the center and the total distance traveled by rat. As shown in Fig. 4A, CRS reduced the frequency of entering the center (8.3 ± 0.60 versus 6.0 ± 0.77), (F = 5.53, *p* = 0.03). A significant decrease of the total distance traveled by rat was observed on the stressed group compared to control (1166 ± 135 versus 497 ± 87) (F = 17,06, *p* = 0.001) (Figure 4B).

### 3.4. ACTH and testosterone level

**Figure 5.**
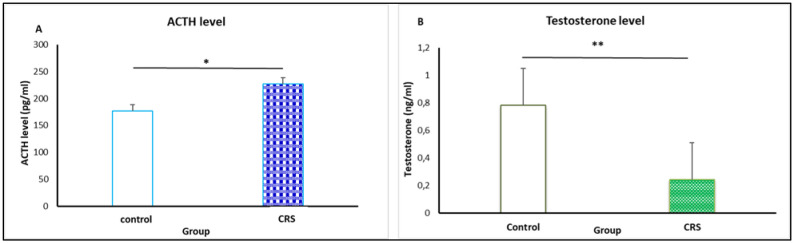
Effect of 2.50 h of restraint stress on ACTH and testosterone level. Data from stressed and control rats (n = 10 per group) are expressed as the mean ± SEM.

As shown in Figure 5A, chronic restraint stress caused significant increase of ACTH level on the stressed group compared to control (226.9 ± 18 versus 176.6 ± 12) (F = 5.27, *p* = 0.034). A significant decrease in testosterone level as compared with the control (0.78 ± 0.12 versus 0.24 ± 0.045), (F =5.53, *p* = 0.002). (Figure 5B).

## 4. Discussion

Animal models are an invaluable instrument for studying aetiology, progress and treatment of depression in a controlled environment. Regarding focus to physical stress effects of on male reproductive health, evidently it is an attractive and valuable field for study. Restricting the free movement of an animal like restraint stress is a method used to induce physiological responses as well as that is primarily a psychological stressor.

The popularity of this stressor method is due to its simplicity, relatively less time, cost and technical requirements. The researchers aimed to provide a clear understanding of the neural mechanisms underlying their etiology. That's why, they have been tailored different parametric manipulation of restraint stress model, such as duration of restraint, number of restraint sessions, time between restraint sessions, and water intake prior to restraint [24].

This study evaluated the validity of 2.5 h durations of CRS in rodent models by analyzing effects on anhedonic-like and masculine sexual behavior. After undergoing CRS for at least 14 days, animals showing a decreased responsiveness to sucrose consumption in the stressed group compared to the control one. Rodents naturally avidly consume drink sweet liquids when given a free choice of two bottles with separate access to sucrose solution and regular water [25],[26].

Similarly, anxiolytic like behavior was correlated with the loss of interesting behavior during SPT in our rats. CRS may induce abnormal behavioral responses in animals as a result of disrupting within the hypothalamic pituitary-adrenal (HPA) axis and suppress the production of new neurons in the hippocampus [27]. Besides that, damage or atrophy to hippocampal CA3 pyramidal cells contributed to this type of stress [28]. A comparative study demonstrated that increasingly severe movement restrictions led to greater behavioral stress responses (29). Although, our data showed that rat exposed to CRS developed anxiety-like behavior. A decreased frequency of entering the center, as well as the total distance traveled in the OFT were shown to be consistent with prior investigations [30],[31]. That may be due to poorer adaption to new environment and increased anxiety-like behaviors rather than impaired physical ability. Thus, an exploratory behavior of rat in new environments, adaptation to the environment and anxiety are more often observed in the first 5 min of the OFT; animals with poor adaptability have notable decrease in the total distance they traveled [32],[33].

Taken together, the findings in the present experiment demonstrated that CRS led to reduced body weight in stress group compared to control in consistent with previous studies [34]. Furthermore, many studies focused on the impact of stress or various stressors on testicular hormone production [35],[36].

In This current study, chronic restraint stress showed significant decrease in plasma level of testosterone, and it has been reported that chronic restraint stress induces to rats a decrease of testosterone responses and a significant anxiety disorder [37]. Consistently, human studies have shown that anhedonia symptoms are associated with under low plasma testosterone levels, in patients with depression [38],[39]. Meanwhile, similar studies have investigated whether stress induced weight loss in rat, chronic restraint stress played a negative effect on testosterone hormone level [40].

To further elucidate the reasons for the reduction in testosterone, previous studies indicated that stress-induced suppression of the hypothalamic-pituitary-gonad (HPG) axis could be mediated by CRF and endogenous opioids, particularly-endorphins, which have been shown to be released from the hypothalamus in response to stress [41]. It is assumed that, CRF and -endorphins have been shown to inhibit LH-RH release from the hypothalamus, LH release from the pituitary, and testosterone synthesis directly in Leydig cells, all of which have effects on the HPG axis. Thus, decreasing testosterone levels in the blood circulation [42].

Morever, in our study, plasma ACTH levels were increased by repeated restraint stress. Thus, we suggest that the increased ACTH circulating levels after restraint stress could be due to a continuous stress state. This increased might affect discrepancies in body weight and induced important changes in behavioral responses in a direct or indirect manner. The increased pattern of ACTH circulating levels may affect the testosterone levels.

Furthermore, the precise mechanism by which stress affects HPA axis as well as HPG axis is not well understood. This antagonistic relationship between both these axes has been proposed to underlie the inhibition of reproductive function due to stress.

Some studies has a correlation between the levels of testosterone and corticosterone in rat [43]. Regardless, in this study, we evaluated the effects of CRS 2.50 h for 14 days on neurobehavior that are accompanied by changes in ACTH and testosterone levels.

There was big heterogeneity between studies on the effects of the CRS protocol, especially the low testosterone level, as a result of many agents, including the animal strains, animal sex, CRS protocol and SPT protocols (e.g., water and food deprivation period, sucrose concentrations, and test period). As well as, circadian rhythm and restraint placement are important factors in CRS protocols that should not be taken in consideration.

## 5. Conclusions

As an advantage of our approach, we were able to triplicate findings of increased ACTH circulating, decreased testosterone levels and maladaptive behaviors among altered neural activity, as an addressing key of challenges in HPG axis. Despite an abundance of experimental and clinical data, the exact pathway between stress-induced hyperactivation of the HPA axis and corresponding attenuation of the HPG axis remains unknown.

## References

[1] Smith KE, Pollak SD (2020). Early life stress and development: potential mechanisms for adverse outcomes. J Neurodevel Disord.

[2] Shoji H, Miyakawa T (2020). Differential effects of stress exposure via two types of restraint apparatuses on behavior and plasma corticosterone level in inbred male BALB/cAJcl mice. Neuropsychopharmacol Rep.

[3] Newsom R (2020). Depression and Sleep. Sleep Foundation.

[4] Guy-Evans O (2021). Hypothalamic-Pituitary-Adrenal Axis. Stress Response HPA Axis.

[5] Sapolsky RM, Romero LM, Munck AU (2000). How do glucocorticoids influence stress responses? Integrating permissive, suppressive, stimulatory, and preparative actions. Endocr Rev.

[6] Heck AL, Handa RJ (2019). Sex differences in the hypothalamic–pituitary–adrenal axis' response to stress: an important role for gonadal hormones. Neuropsychopharmacol.

[7] Buynitsky T, Mostofsky DI (2009). Restraint stress in biobehavioral research: recent developments. Neurosci Biobehav Rev.

[8] Paré WP, Glavin GB (1986). Restraint stress in biomedical research: a review. Neurosci Biobehav Rev.

[9] Hjollund N, Bonde J, Henriksen T (2004). Reproductive effects of male psychologic stress. Epidemiology.

[10] El-Naggar HAEM, El-Safty FENA, El-mehi AE (2020). Effect of Chronic Stress on The Testis of The Adult Male Albino Rat and The Role of Ginger. Egypt J Hosp Med.

[11] Wingfield J, Sapolsky R (2003). Reproduction and resistance to stress: when and how. J Neuroendocrinol.

[12] Oduwole OO, Peltoketo H, Huhtaniemi IT (2018). Role of follicle-stimulating hormone in spermatogenesis. Front Endocrinol.

[13] Chatterjee A, Rajikin MH, Chatterjee R (2006). Stress and how it affects reproduction. Biomed Res.

[14] Seewoo BJ, Hennessy LA, Feindel KW (2020). Validation of chronic restraint stress model in rats for the study of depression using longitudinal multimodal MR imaging. bioRxiv.

[15] Ulloa JL, Castañeda P, Berríos C (2010). Comparison of the antidepressant sertraline on differential depression-like behaviors elicited by restraint stress and repeated corticosterone administration. Pharmacol Biochem Behav.

[16] Díaz A, León P, Conde C (2010). Evaluación del efecto de la Administración Aguda de Bromocriptina Sobre el Consumo de Sacarosa en Ratas Sometidas a Aislamiento Social.

[17] Alvarez D (2015). Anhedonia en Perros: Efecto del Estrés Sobre la Preferencia Frente a Sacarosa. Universidad de Chile.

[18] Acero-Castillo MC, Ardila-Figueroa MC, de Oliveira SB (2021). Anhedonic type behavior and anxiety profile of Wistar-UIS rats subjected to chronic social isolation. Front Behav Neurosci.

[19] Klein DF (1974). Endogenomorphic depression: a conceptual and terminological revision. Arch Gen Psychiatry.

[20] Vidal J (2014). Open field modifications needed to measure, in the mouse, exploration-driven ambulation and fear of open space. Anuario de psicología/ UB J psychol.

[21] Xu XE, Liu L, Wang Y (2019). Caspase-1 inhibitor exerts brain-protective effects against sepsisassociated encephalopathy and cognitive impairments in a mouse model of sepsis. Brain Behav Immun.

[22] Denenberg VH (1969). Open-field behavior in the rat: What does it mean?. Ann N Y Acad Sci.

[23] Engvall E, Perlman P (1971). Enzyme-linked immunosorbent assay (ELISA). Quantitative assay of immunoglobulin G. Immunochem.

[24] Servatius RJ, Salameh G, Coyle KM (2007). *Restraint Stress**, Encyclopedia of Stress.

[25] Goshen I, Kreisel T, Ben-Menachem-Zidon O (2008). Brain interleukin-1 mediates chronic stress-induced depression in mice via adrenocortical activation and hippocampal neurogenesis suppression. Mol Psychiatry.

[26] Liu MY, Yin CY, Zhu LJ (2018). Sucrose preference test for measurement of stressinduced anhedonia in mice. Nat Protoc.

[27] Mao Y, Xu Y, Yuan X (2022). Validity of chronic restraint stress for modeling anhedonic-like behavior in rodents: a systematic review and meta-analysis. J Int Medl Res.

[28] Wang Q, Timberlake IIMA, Prall K (2017). The recent progress in animal models of depression. Prog Neuro-Psychopharmacol Biol Psychiatry.

[29] Ampuero E, Luarte A, Santibanez M (2015). Two Chronic Stress Models Based on Movement Restriction in Rats Respond Selectively to Antidepressant Drugs: Aldolase C As a Potential Biomarker. Int J Neuropsychopharmacol.

[30] Chai C, Jin B, Yan Y (2021). Anti-depressant effect of Zhi-zi-chi decoction on CUMS mice and elucidation of its signaling pathway. J Ethnopharmacol.

[31] Zhong F, Liu L, Wei JL (2019). Brain-derived neurotrophic factor precursor in the hippocampus regulates both depressive and anxiety-like behaviors in rats. Front Psychiatry.

[32] Morgan JA (2018). The effects of aerobic exercise on depression-like, anxiety-like, and cognition-like behaviours over the healthy adult lifespan of C57BL/6 mice. Behav Brain Res.

[33] Walsh RN, Cummins RA (1976). The open-field test: a critical review. Psychol Bull.

[34] Fee C, Prevot T, Misquitta K (2020). Chronic stress exacerbates acute stress-induced neuronal activation in the anterior cingulate cortex and ventral hippocampus that correlates with behavioral deficits in mice. bioRxiv.

[35] Orr TE, Mann DR (1990). Effects of restraint stress on plasma LH and testosterone concentrations, Leydig cell LH/hCG receptors, and in vitro testicular steroidogenesis in adult rats. Horm Behav.

[36] SAFYA EE, RADWA M (2018). Effect of Visfatin on Testosterone Hormone Level in Chronic Restraint Male Albino Rats. Med J Cairo Univ.

[37] Bagheri Y, Fathi E, Maghoul A (2021). Effects of Achillea tenuifolia Lam. hydro-alcoholic extract on anxiety-like behavior and reproductive parameters in rat model of chronic restraint stress. Hum Exp Toxicol.

[38] McHenry J, Carrier N, Hull E (2014). Sex differences in anxiety and depression: role of testosterone. Front Neuroendocrinol.

[39] Määttänen I, Gluschkoff K, Komulainen K (2021). Testosterone and specific symptoms of depression: Evidence from NHANES 2011–2016. Compr Psychoneuroendocrinol.

[40] Huang Y, Wen J, Chen G (2021). Role and Mechanism of Chronic Restraint Stress in Regulating Energy Metabolism and Reproductive Function Through Hypothalamic Kisspeptin Neurons. J Endoc Soc.

[41] Vaccarino AL, Kastin AJ (2000). Endogenous Opiates. Pept.

[42] Damegh MA (2014). Stress-induced changes in testosterone secretion in male rats: role of oxidative stress and modulation by antioxidants. Open J Anim Sci.

[43] Retana-Márquez S, Bonilla-Jaime H, Vazquez-Palacios G (2003). Changes in masculine sexual behavior, corticosterone and testosterone in response to acute and chronic stress in male rats. Horm Behav.

